# Impact of Hypoxia-Inducible Factor Prolyl Hydroxylase Inhibitor on Heart Failure with Preserved Ejection Fraction

**DOI:** 10.3390/medicina57121319

**Published:** 2021-12-01

**Authors:** Teruhiko Imamura, Masakazu Hori, Shuhei Tanaka, Koichiro Kinugawa

**Affiliations:** The Second Department of Internal Medicine, University of Toyama, Toyama 9300194, Japan; masahori6059@yahoo.co.jp (M.H.); stanaka@med.u-toyama.ac.jp (S.T.); kinugawa0422@gmail.com (K.K.)

**Keywords:** heart failure, chronic kidney disease, reverse remodeling

## Abstract

Hypoxia-inducible factor prolyl hydroxylase (HIF-PH) inhibitor is a recently introduced oral medication to treat renal anemia, but its clinical implication in patients with heart failure, particularly heart failure with preserved ejection fraction (HFpEF), remains unknown. We had a 91-year-old woman with HFpEF who was admitted to our institute to treat her worsening heart failure. She initiated HIF-PH inhibitor daprodustat to treat her renal anemia (hemoglobin 8.8 g/dL and estimated glomerular filtration ratio 15.6 mL/min/1.73 m^2^). Following a 6-month treatment with daprodustat, hemoglobin increased up to 10.4 g/dL, left ventricular mass index decreased from 107 g/m^2^ to 88 g/m^2^, and plasma B-type natriuretic peptide decreased from 276 pg/mL to 122 pg/mL, despite doses of other medications remaining unchanged. HIF-PH inhibitors might be a promising tool to ameliorate renal anemia and facilitate cardiac reverse remodeling in patients with HFpEF.

## 1. Introduction

A deep association among anemia, heart failure, and chronic kidney disease is receiving great concern, and is known as “cardio-renal-anemia syndrome” [1], in which the existence of one disease worsens others by forming a vicious cycle. Of note, the existence of anemia is an independent risk factor of mortality in patients with heart failure [2], whereas anemia progresses in patients with heart failure via dilution, chronic inflammation and cardiac cachexia, and bone marrow suppression [3]. Anemia is considered to be a good therapeutic target to terminate such a vicious cycle, but the clinical benefit of aggressive interventions to anemia using transfusion or erythropoiesis-stimulating agent in patients with heart failure remains controversial.

Recently, hypoxia-inducible factor prolyl hydroxylase (HIF-PH) inhibitor, which is orally administered and increases erythropoietin and other related factors by stabilizing HIF-alpha, has become clinically available to treat renal anemia [4]. A phase III trial demonstrated noninferiority of HIF-PH inhibitor to conventional erythropoietin stimulating agent in improving renal anemia [5]. However, the clinical implication of HIF-PH inhibitors in ameliorating renal anemia and improving mortality and morbidity in patients with heart failure and chronic kidney disease remains unknown. 

## 2. Case Report

### 2.1. On Admission

A 91-year-old woman with a history of transcatheter aortic valve replacement two years ago was admitted to our institute complaining of bilateral leg edema and dyspnea on exertion (New York Heart Association functional class III). She had received bisoprolol 0.625 mg/day, olmesartan 10 mg/day, tolvaptan 3.75 mg/day, and azosemide 60 mg/day as well as rivaroxaban 10 mg/day. She did not have any history of malignancy. 

**Figure 1:**
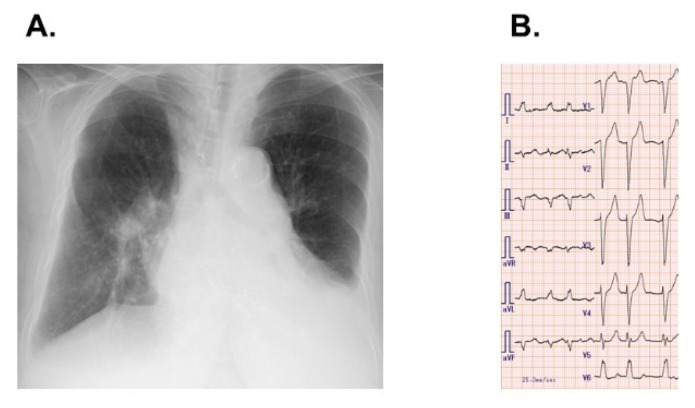
Chest X-ray (**A**) and electrocardiogram (**B**) on admission.

Body height was 143 cm and body weight was 54 kg. Blood pressure was 113/47 mmHg and pulse rate was 71 bpm. Chest X-ray showed mild cardiomegaly, dilatation of pulmonary artery, and mild pleural effusion (Figure 1A). An electrocardiogram showed heart rate 99 bpm, atrial fibrillation, and complete left bundle branch block (Figure 1B). Hemoglobin was 8.5 g/dL, erythropoietin was 41.1 mIU/mL, ferritin was 303 ng/mL, transferrin saturation was 30.3%, and reticulocyte was 0.3%. Fecal occult blood test was negative. Serum lactate dehydrogenase level was 143 IU/L. Estimated glomerular filtration ratio was 18 mL/min/1.73 m^2^ and Plasma B-type natriuretic peptide was 414 pg/mL. 

### 2.2. In-Hospital Course

Following the admission, intravenous furosemide and potassium canrenoate were administered. The dose of tolvaptan was increased to 7.5 mg/day and that of azosemide was decreased to 30 mg/day to prevent hypovolemia and preserve renal function. Daprodustat 2 mg/day was initiated to treat renal anemia before the index discharge. 

**Figure 2:**
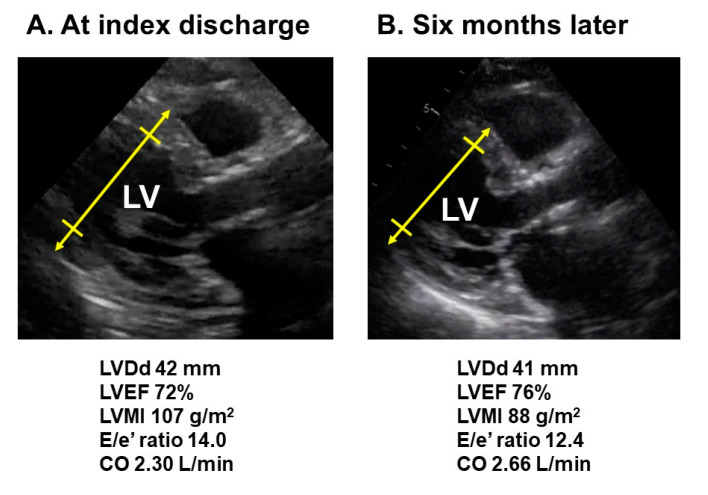
Long-axis views of transthoracic echocardiography obtained at index discharge (**A**) and six-month follow-up (**B**); LV, left ventricle; LVDd, left ventricular end-diastolic diameter; LVEF, left ventricular ejection fraction; LVMI, left ventricular mass index; CO, cardiac output.

At index discharge, body weight was 48 kg, hemoglobin was 8.8 g/dL, estimated glomerular filtration ratio was 15.6 mL/min/1.73 m^2^, and plasma B-type natriuretic peptide was 276 pg/mL. Transthoracic echocardiography showed left ventricular end-diastolic diameter of 42 mm, left ventricular ejection fraction of 72%, E/e’ ratio of 14.0, and left ventricular mass index of 107 g/m^2^ (Figure 2A). Cardiac output was 2.30 L/min. 

### 2.3. Post-Discharge Course

**Figure 3:**
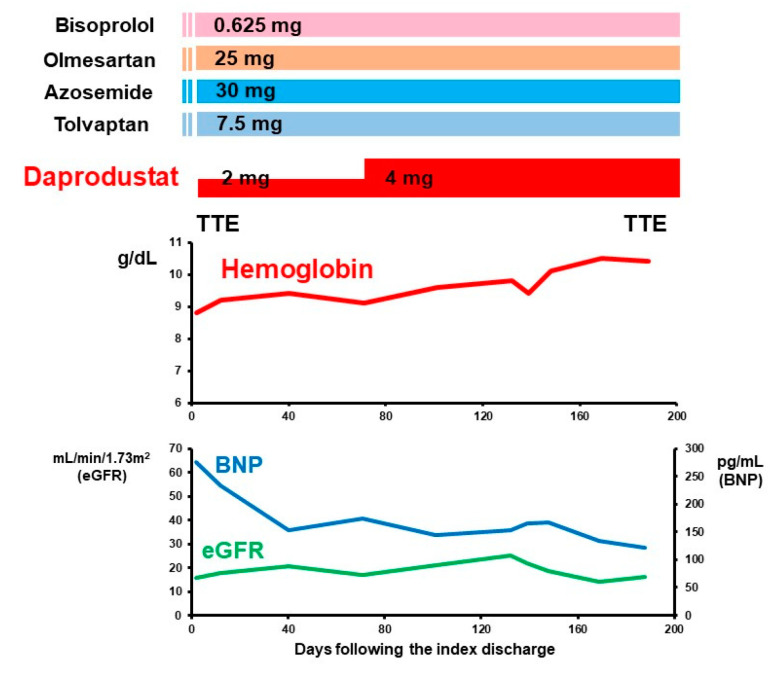
Six-month time course following the index discharge. TTE, transthoracic echocardiography; BNP, B-type natriuretic peptide; eGFR, estimated glomerular filtration ratio.

Following the index discharge, daprodustat was continued for six months without any adverse events, including thromboembolism. Systolic blood pressure remained around 110 mmHg. Medications remained unchanged except for daprodustat, which was up-titrated to 4 mg/day at 70 days following the index discharge (Figure 3).

During the 6-month follow-up period, the hemoglobin level increased gradually from around 9.0 g/dL up to 10.0 g/dL. Plasma B-type natriuretic peptide decreased from around 250 pg/mL down to 150 pg/mL. Renal function remained unchanged. At 6-month follow-up, left ventricular mass index decreased to 88 g/m^2^ and cardiac output increased to 2.66 L/min (Figure 2B). Ferritin was 216 ng/mL. The degree of tricuspid regurgitation remained trace. New York Heart Association functional class improved to II.

## 3. Discussion

### 3.1. Differential Diagnosis of Renal Anemia

Before the definite diagnosis of renal anemia and initiation of HIF-PH inhibitors, a differential diagnosis should be highly encouraged particularly in patients with heart failure, who have various and sometimes complex and multiple causes of anemia [6].

Iron deficiency is a major cause of anemia, which was excluded by several laboratory data including ferritin and transferrin saturation. We cannot deny the contribution of dilutional anemia given the existence of systemic congestion. Hemolytic anemia was denied by a normal level of lactate dehydrogenase, although she had received aortic valve replacement. Chronic inflammation due to cardiac cachexia would have contributed to the progression of anemia via suppression of erythropoietin synthesis and refractoriness to erythropoietin in the bone marrow [3]. We considered that a major contributor of anemia in this patient was renal anemia given the existence of chronic kidney disease and insufficiently increased erythropoietin level, and we initiated HIF-PH inhibitor.

### 3.2. Impact of HIF-PH Inhibitor on Cardiac Reverse Remodeling

The impact of aggressive correction of anemia in patients with heart failure is controversial. Several randomized control trials using erythropoietin-stimulating agents did not reduce cardiovascular events or were rather harmful [7,8]. Aggressive intervention using erythropoietin-stimulating agents is considered to increase the risk of thromboembolic events. Hereby, the impact of HIF-PH inhibitors on heart failure patients has not yet been investigated thus far.

In this patient, although other heart failure medications remained unchanged, both left ventricular mass index and plasma B-type natriuretic peptide level improved considerably over 6 months of daprodustat therapy, with hemoglobin increased and maintained around 10 g/mL. Against the existence of heart failure-related chronic inflammation [3], daprodustat might be able to also correct renal anemia in patients with heart failure. Furthermore, corrected anemia might decrease the afterload, unload left heart, and facilitate cardiac reverse remodeling.

On the contrary, several experimental studies have raised the caution that HIF-PH inhibitors might rather worsen pulmonary hypertension and heart failure. Further studies are warranted to derive any conclusions for the long-term use of HIF-PH inhibitors on those with cardiovascular diseases.

The use of B-type natriuretic peptide as a surrogate might require a specific caution during HIF-PH inhibitors therapy. The stabilization of HIF-1α might directly stimulate B-type natriuretic peptide induction. A decrease in B-type natriuretic peptide still would be observed in patients with extensive improvement in heart failure, as our patient.

The patient did not experience any associated complications, but an ideal hemoglobin target, at which patients can enjoy maximum reverse remodeling and minimum mortality and morbidity, remains the next concern. The impact of HIF-PH inhibitors on other clinical outcomes including exercise capacity and end-organ function also remains a future concern. The applicability of our observation using daprodustat on other HIF-PH inhibitors is uncertain.

## 4. Conclusions

HIF-PH inhibitors might be a promising tool to ameliorate renal anemia and facilitate cardiac reverse remodeling in patients with HFpEF, although further studies are warranted to establish optimal therapeutic strategy using HIF-PH inhibitors among such a cohort.

## Data Availability

Data are available from the corresponding author upon reasonable request.
